# γδ T Cell-Mediated Antibody-Dependent Cellular Cytotoxicity with CD19 Antibodies Assessed by an Impedance-Based Label-Free Real-Time Cytotoxicity Assay

**DOI:** 10.3389/fimmu.2014.00618

**Published:** 2014-12-02

**Authors:** Ursula Jördis Eva Seidel, Fabian Vogt, Ludger Grosse-Hovest, Gundram Jung, Rupert Handgretinger, Peter Lang

**Affiliations:** ^1^Department of General Paediatrics, Oncology/Haematology, University Children’s Hospital Tübingen, Tübingen, Germany; ^2^Department of Immunology, Interfaculty Institute for Cell Biology, University of Tübingen, Tübingen, Germany; ^3^Partner Site Tübingen, German Cancer Consortium (DKTK), German Cancer Research Center (DKFZ), Tübingen, Germany; ^4^SYNIMMUNE GmbH, Tübingen, Germany

**Keywords:** γδ T cell expansion, ADCC, tumor immunotherapy, therapeutic antibodies, xCELLigence system

## Abstract

γδ T cells are not MHC restricted, elicit cytotoxicity against various malignancies, are present in early post-transplant phases in novel stem cell transplantation strategies and have been shown to mediate antibody-dependent cellular cytotoxicity (ADCC) with monoclonal antibodies (mAbs). These features make γδ T cells promising effector cells for antibody-based immunotherapy in pediatric patients with B-lineage acute lymphoblastic leukemia (ALL). To evaluate combination of human γδ T cells with CD19 antibodies for immunotherapy of B-lineage ALL, γδ T cells were expanded after a GMP-compliant protocol and ADCC of both primary and expanded γδ T cells with an Fc-optimized CD19 antibody (4G7SDIE) and a bi-specific antibody with the specificities CD19 and CD16 (N19-C16) was evaluated in CD107a-degranulation assays and intracellular cytokine staining. CD107a, TNFα, and IFNγ expression of primary γδ T cells were significantly increased and correlated with CD16-expression of γδ T cells. γδ T cells highly expressed CD107a after expansion and no further increased expression by 4G7SDIE and N19-C16 was measured. Cytotoxicity of purified expanded γδ T cells targeting CD19-expressing cells was assessed in both europium-TDA release and in an impedance-based label-free method (using the xCELLigence system) measuring γδ T cell lysis in real-time. Albeit in the 2 h end-point europium-TDA release assay no increased lysis was observed, in real-time xCELLigence assays both significant antibody-independent cytotoxicity and ADCC of γδ T cells were observed. The xCELLigence system outperformed the end-point europium-TDA release assay in sensitivity and allows drawing of conclusions to lysis kinetics of γδ T cells over prolonged periods of time periods. Combination of CD19 antibodies with primary as well as expanded γδ T cells exhibits a promising approach, which may enhance clinical outcome of patients with pediatric B-lineage ALL and requires clinical evaluation.

## Introduction

Pediatric B-lineage acute lymphoblastic leukemia (ALL) is the most common childhood malignancy and the leading cause of cancer-related death during childhood (1). Improvement of chemotherapeutic protocols as well as application of allogeneic stem cell transplantation (SCT) in relapsed or refractory patients has improved outcome tremendously. Graft manipulation strategies have evolved from CD34-positive selection over CD3/CD19 depletion to TCRαβ/CD19 depletion, leaving γδ T cells in the graft and which is currently applied in phase I/II clinical studies (2–4). In contrast to αβ T cells, γδ T cells are not restricted by MHC molecules, which makes them unlikely to elicit graft versus host disease (GvHD) based on HLA alloreactivity (5). This feature makes γδ T cells potent effector cells not only during early lymphopenic post-transplant phase but also in cell-based immunotherapy after SCT.

Immunotherapy with CD20-specific monoclonal antibodies (mAbs) Rituximab and Ofatumumab has shown promising clinical results in B-cell malignancies. However, CD20 is expressed in <50% of pediatric B-lineage ALLs, thus, limiting the potential use of CD20 antibodies to a minor patient cohort (6, 7). CD19, another signature B-cell antigen, is expressed from early B-cell development onward and, thus, expressed on all B-lineage ALLs making it a well suited target antigen (8, 9). Several CD19 antibodies are currently in pre-clinical and early clinical evaluation (7–12). Main effector function of these mAbs is antibody-dependent cellular cytotoxicity (ADCC), which is mediated by the activating low-affinity receptor FcγRIIIa (type III receptor for IgG; CD16), which binds the Fc portion of human antibodies of the subclasses IgG1 and IgG3. Engagement of CD16 induces a potent activating signal, which overcomes inhibitory signals and results in one or more of the effector functions ADCC, cytokine response, and phagocytosis (13). ADCC is mediated by the release of cytotoxic granules containing perforin and granzyme leading to the lysis of target cells. The relevance of ADCC *in vivo* has been underlined by a recent study showing improved clinical response in patients showing higher capacity for ADCC *in vitro* (14). CD16 is highly expressed by natural killer (NK) cells and by other hematopoietic cells including macrophages and granulocytes.

γδ T cells share several surface antigens with NK cells, including NKG2D, ULBP, CD56, and CD16 (15). CD16-expression of circulating Vγ9Vδ2 T lymphocytes may be induced by activating γδ T cells with phosphoantigens and this distinct subset of effector cells has been shown to be highly cytolytic against tumor cells upon activation via CD16 (16, 17). ADCC induced by CD16-expressing γδ T cells has been shown for therapeutic antibodies as Rituximab and Trastuzumab (18, 19).

Besides second generation mAbs as chimerized antibody Rituximab and humanized antibody Trastuzumab, several third-generation antibodies have been developed in order to further enhance ADCC *in vivo* and, thus, improving clinical efficacy (20). The main approaches to optimize FcγRIIIa binding by enhancing the affinity of mAbs developed in recent years, were molecular modifications in the Fc domain of mAbs leading to amino acid substitutions (21–23), modifying Fc-linked glycosylation (24–26) and replacement of the reactive Fc portion by a binding domain for CD16 (27). For treatment of acute myeloid leukemia (AML) several of these third-generation constructs are currently under pre-clinical and early clinical investigation and have been shown to mediate higher ADCC than their unmodified counterparts (28–30).

The standard techniques to determine the antibody-independent cytotoxicity (AIC) and ADCC *in vitro* include ^51^chromium release assays, Europium-TDA assays, [(3)H] thymidine incorporation assays, MTT assays, and flow cytometry-based CD107a-degranulation assays (31–35). However, those methods share various limitations including the labeling of cells and that they can only be readily performed as end-point assays, thereby lacking the information required for kinetic studies (36). Recent studies reported on the deployment of a novel label-free electrical impedance-based assay allowing the dynamic detection of AIC and ADCC and suggest several advantages compared to other established killing assays. This technique, based on the continuous assessment of electrical impedance, has been validated for the assessment of NK cell AIC and ADCC and antigen-specific T-cell-mediated cytotoxicity and deployed for the assessment of γδ T cell-mediated cytotoxicity with bi-specific antibodies binding CD3 and Vγ9 on γδ T cells, respectively (36–38). Impedance to an electric current is increased by the isolating properties of the cell body, when adherent tumor cells attach to electrodes on the bottom of multi-well plates. Killing of these tumor cells results in detachment or disintegration, reducing the electrical impedance that can be measured by the xCELLigence system (36).

Here, we not only show that primary as well as expanded γδ T cells mediate ADCC with an Fc-optimized CD19 antibody and a CD19–CD16 bi-specific construct but present a label-free impedance-based method, facilitating the detection of γδ T cell lysis kinetics over prolonged periods of time.

## Material and Methods

### Cells and culture conditions

PBMC from leukocytes of thrombapheresises of healthy blood donors and leukemic blasts were isolated by density gradient centrifugation using Biocoll Separating Solution (Biochrom, Berlin, Germany). Healthy donor samples were kindly provided by the Institute for Clinical and Experimental Transfusion Medicine at Tübingen University after obtaining written informed consent. Primary leukemic blasts were obtained from a patient with common-ALL. Over 90% of bone marrow cells were positive for CD10/CD34/CD19 as determined by flow cytometry. PBMC, leukemic blasts, and pediatric B-lineage ALL cell line SEM (ACC 546, DSMZ, Braunschweig, Germany) were cultured in IMDM (Lonza, Basel, Switzerland), breast adenocarcinoma cell line MCF-7 (ACC 115), and B-lineage ALL cell line NALM-6 (ACC 128) were kept in EMEM and RPMI 1640 (Biochrom), respectively. All media were supplemented with 10% fetal calf serum or pooled human AB serum (Invitrogen, Karlsruhe, Germany), 100 U/ml penicillin, 100 μg/ml streptomycin, 1 mM sodium pyruvate, and 2 mM L-glutamine (all reagents Biochrom).

### MCF-7 transfectant

Full-length cDNA of human CD19 (GenBank no. BC006338.2) was purchased from source Bioscience (Berlin, Germany) and cloned into the expression vector pGH-1. MCF-7 cells were transfected by electroporation (230 V, 975 μF) and CD19 expressing clones were selected and sorted by flow cytometric analysis. Stable CD19 expression of transfected MCF-7 (termed MCF-7-CD19tm) was verified over course of culture. Medium of selected clones was supplemented with 1 mg/ml G418-BC sulfate (Biochrom).

### Antibodies and flow cytometry

Initial chimerization and Fc-optimization of χ4G7 and 4G7SDIE, respectively, was described previously (28). For a good manufacturing practice (GMP)-compliant production, antibody genes, codon-usage optimized for CHO expression, were inserted into the expression vector pGH-1 and used for serum-free transfection of CHO cells. The antibody 4G7SDIE was then produced by transfected CHO cells and purified in GMP-compliant clean rooms using disposable technology including a 100-l biowave reactor (Sartorius, Goettingen, Germany) for fermentation and an ÄKTA ready system for purification by protein A, ion exchange, and hydrophobic interaction chromatography (MabSelect SuRe and Capto-Adhere columns, GE Healthcare, Munich, Germany). N19-C16 (CD19–CD16) and N19-CU (CD19–CD3) were generated in a bi-specific format, termed Fabsc. This bi-specific format contains the CD19 antibody (clone 4G7) as a Fab fragment, which is linked by an Fc-attenuated CH2 domain to a C-terminal single-chain Fv fragments derived from the CD16 antibody (clone 3G8) and the CD3 antibody (clone UCHT1), respectively (Durben et al., manuscript submitted). The constructs were produced by transfected Sp2/0 cells and purified by affinity chromatography with κ-select (GE Healthcare).

CD107a-APC, CD56-PE/Cy7, CD45-APC/Cy7, CD3-PerCP, IFNγ-BV711, TNFα-PB, CD16-AF700, CD4-BV421, CD8-APC/Cy7, and isotype control antibodies were purchased from Biolegend (San Diego, CA, USA). TCRγδ-FITC, CD19-PE, CD56-PE, and anti-human CD19 antibody (clone 4G7) were purchased from BD Biosciences (Heidelberg, Germany) and LIVE/DEAD Fixable Aqua and Yellow Dead Stain Kits from Invitrogen. All antibodies were incubated with cells for 20 min at 4°C. Quantitative analysis was performed with QIFIKIT (Dako, Hamburg, Germany) according to the manufacturers’ recommendations. Cells were analyzed with a FACSCalibur or a LSRII and sorted with a FACSJazz (BD Biosciences).

### CD107a-degranulation assay

Percentage of γδ T cells (CD3^+^, TCRγδ^+^) was determined by flow cytometry and donors with γδ T cells >1.5% were selected for CD107a assays. Equal cell numbers of PBMC and NALM-6 or SEM were incubated with 1 μg/ml 4G7SDIE or N19-C16, 2 μM GolgiStop (BD Biosciences), 10 μg/ml Brefeldin A (Sigma, Steinheim, Germany) and CD107a-APC overnight at 37°C, 5% CO_2_ in supplemented IMDM. Subsequently, PBMC were stained for surface and intracellular markers and analyzed by flow cytometry.

### Expansion of γδ T cells

PBMC were seeded at 1.5 × 10^6^ per well in 24-well plates and cultured in supplemented IMDM containing 100 IU/ml of recombinant human IL-2 (rhIL-2) (Novartis, Basel, Switzerland) and 400 nM zoledronate (Hexal, Holzkirchen, Germany). Every 2–3 days medium containing 100 IU/ml rhIL-2 and 400 nM zoledronate was added. After 12–14 days of culture expanded populations were positively selected using a Hapten-modified anti TCR-γδ antibody and FITC-conjugated anti-Hapten MicroBeads with the autoMACS system (Miltenyi Biotec, Bergisch Gladbach, Germany). Purity of the isolated populations was determined by flow cytometric analysis and isolated cells were incubated with 400 IU/ml rhIL-2 overnight prior to functional assays. After 24 h isolated γδ T cells had lost their FITC-labeling and restored TCRγδ surface expression (data not shown).

### Real-time cytotoxicity assay (xCELLigence assay)

The cytolytic potential of expanded and isolated γδ T cells was analyzed in a real-time cytotoxicty assay with an xCELLigence RTCA SP instrument (ACEA Biosciences, San Diego, CA, USA). In each well 5 × 10^3^ MCF-7-CD19tm cells were seeded. After 20–24 h expanded γδ T cells and 1 μg/ml 4G7SDIE and N19-C16 were added, respectively. Cell viability was monitored every 15 min for 48 h. Cell indexes (CIs) were normalized to CI of the time-point when γδ T cells were added and specific lysis was calculated in relation to the control cells lacking any effector γδ T cells.

### Europium-TDA cytotoxicity assay

The cytolytic activity of expanded and isolated γδ T cells was analyzed in a 2 h-DELFIA EuTDA cytotoxicity assay (PerkinElmer, Waltham, MA, USA) according to the manufacturers recommendations and as described previously (39). Briefly, cryopreserved primary B-lineage ALL blasts were labeled with the fluorescence enhancing ligand BATDA for 60 min at 37°C. After five wash cycles 5 × 10^3^ target cells per well were seeded and γδ T cells and 1 μg/ml 4G7SDIE and N19-C16 were added, respectively. After co-culture of 2 h, 20 μl of supernatant was mixed with 200 μl DELFIA Europium Solution and after 15 min fluorescence of Europium-TDA chelates was quantified using a VICTOR multi label reader (Wallac, Turku, Finland). Specific lysis was calculated as follows: % specific lysis = (experimental TDA release − spontaneous TDA release)/(maximum TDA release − spontaneous TDA release) × 100.

### Graphical and statistical analysis

Flow cytometry data were analyzed with FlowJo software (Tree Star, Ashland, OR, USA) and xCELLigence data were analyzed with RTCA Software 1.2 (ACEA Biosciences). Other analyses were performed using GraphPad Prism software (GraphPad Software, La Jolla, CA, USA). Statistical significance was accepted at *p* < 0.05 and is indicated by *(*p* < 0.05), **(*p* < 0.01), ***(*p* < 0.001), and ****(*p* < 0.0001).

## Results

### Expansion of human CD16^+^ γδ T cells

Human γδ T cells, obtained from leukapheresis products from six healthy volunteers, were expanded after a GMP-compliant protocol using 400 nM zoledronate and 100 IU/ml rhIL-2. After 12–14 days of culture γδ T cells were enriched from 2.87% ± 1.48 of PBMC prior to expansion to 42.35% ± 10.85 of cultured cells. A 1.27-fold ±0.49 expansion of total cells and a 22.55-fold ±9.91 expansion of γδ T cells were observed. Percentage of CD16^+^ γδ T cells reached 26.55% ± 6.02 after expansion (Table 1). As described previously, expanded γδ T cells exhibited an continuum of CD16-expression (data not shown) (17, 18).

**Table 1:** **Expansion of human γδ T cells**.

Donor	γδ T cells prior to expansion (%)	Days of expansion	γδ T cells after expansion (%)	CD16^+^ γδ T cells after expansion (%)	Fold-expansion of total cells	Fold-expansion of γδ T cells cells
1	2.24	13	35.9	22.70	0.93	14.84
2	1.60	13	43.8	30.26	1.12	30.66
3	4.51	14	60.6	35.10	2.13	28.59
4	1.20	13	33.8	28.90	0.88	24.84
5	5.18	13	32.0	24.00	1.01	6.22
6	2.50	12	48.0	18.32	1.57	30.13
Mean (SD)	2.87 (±1.61)	13 (±0.63)	42.35 (±10.85)	26.55 (±6.02)	1.27 (±0.49)	22.55 (±9.91)

### Indirect detection of cytotoxicity of primary γδ T cells and CD19-specific antibody constructs against CD19^+^ leukemic cell lines

**Figure 1:**
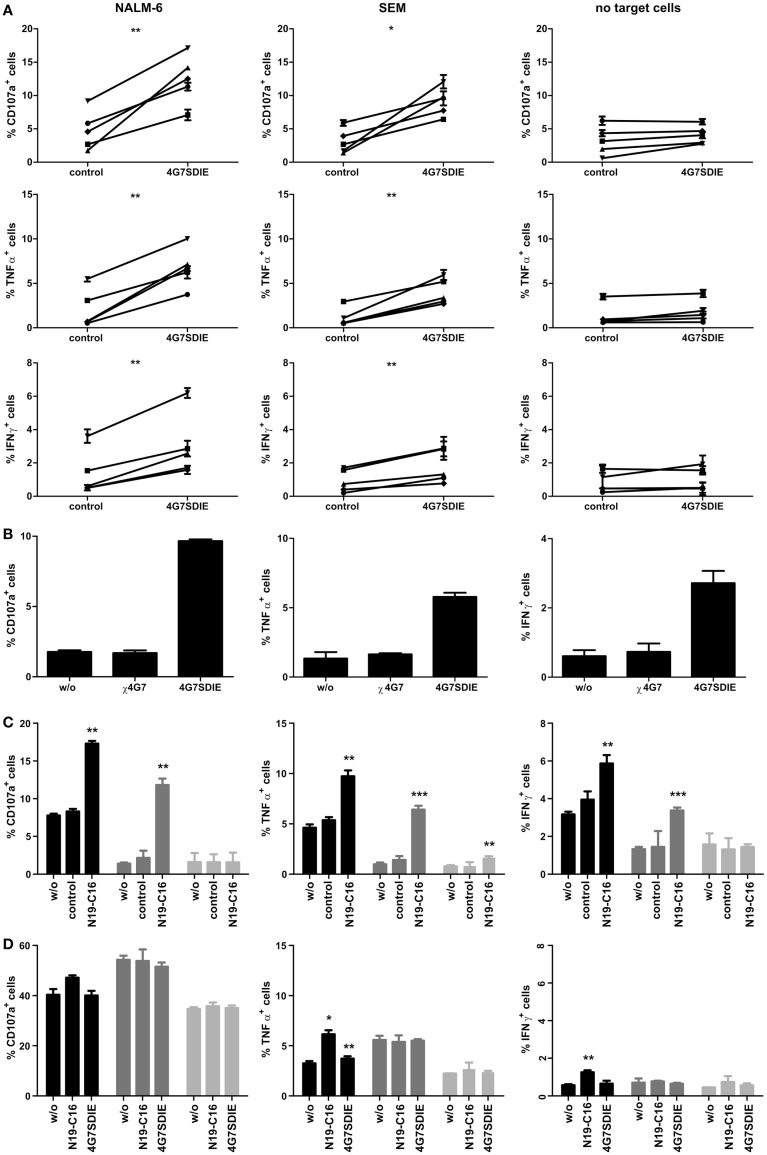
**Indirect assessment of primary and expanded γδ T cell-mediated AIC and ADCC in CD107a-degranulation assays and intracellular cytokine stainings (ICS)**. PBMC **(A–C)** or expanded, isolated, and recovered γδ T cells **(D)** were incubated with equal cell numbers of target cell lines NALM-6 or SEM and antibodies for 16 h. γδ T cell degranulation (CD107a) and cytokine production (TNFα and IFNγ) were detected on viable, single CD45^+^CD3^+^TCRγδ^+^ lymphocytes by extracellular and intracellular staining and flow cytometric analysis. **(A)** PBMC were incubated with or without target cell lines NALM-6 or SEM and 1 μg/ml control antibody or 1 μg/ml 4G7SDIE. Five different donors of 5 independent experiments are shown. **(B)** PBMC were incubated with target cell line NALM-6 and without antibody, 1 μg/ml χ4G7 or 1 μg/ml 4G7SDIE. One representative experiment, of three independent experiments performed, is shown. **(C)** PBMC were incubated with or without (light gray bars) target cell lines NALM-6 (black bars) or SEM (gray bars) and without, 1 μg/ml control antibody or 1 μg/ml N19-C16. Experiments were performed in triplicates. **(D)** Expanded, isolated, and recovered γδ T cells were incubated with or without (light gray bars) target cell lines NALM-6 (black bars) or SEM (gray bars) and without, 1 μg/ml N19-C16 or 1 μg/ml 4G7SDIE. Experiments were performed in triplicates. Error bars represent the standard deviation (SD).

Due to low percentages of primary γδ T cells in PBMC samples (Table 1), isolation of sufficient numbers of γδ T cells in order to perform cytotoxicity assays may be challenging. Hence, an assay detecting the activation of γδ T cells in PBMC samples deploying the degranulation marker CD107a as well as simultaneously measuring cytokine production by intracellular cytokine staining (ICS) for IFNγ and TNFα was established. PBMC samples were incubated with pediatric B-cell precursor leukemia cell lines NALM-6 or SEM and CD19 antibody 4G7SDIE or N19-C16 constructs. CD107a, IFNγ, and TNFα expression of CD3^+^TCRγδ^+^ γδ T cells were determined using flow cytometry. γδ T cells incubated with NALM-6 or SEM and γδ T cells incubated with irrelevant control antibody and NALM-6 or SEM, respectively, displayed low expression of CD107a, IFNγ, and TNFα (Figure 1). However, Fc-optimized CD19 antibody 4G7SDIE enhanced CD107a and TNFα expression of γδ T cells, when incubated with target cell lines, significantly (Figure 1A). IFNγ expression was significantly enhanced as well, though percentages of positive cells remained low. Without adding target cell lines, γδ T cells incubated with 4G7SDIE did not express significantly enhanced levels of CD107a, IFNγ, and TNFα (Figure 1A). The chimerized, unmodified counterpart of 4G7SDIE, χ4G7, did not increase CD107a, IFNγ, and TNFα expression of γδ T cells when incubated with target cell lines NALM-6 (Figure 1B). Bi-specific CD19–CD16 antibody construct N19-C16 induced a significantly enhanced CD107a, IFNγ as well as TNFα expression of γδ T cells, when incubated with target cell lines, as well (Figure 1C).

### Indirect detection of cytotoxicity of expanded γδ T cells and CD19-specific antibody constructs against CD19^+^ leukemic cell lines

γδ T cells were expanded as described and the TCRγδ^+^-cell population was isolated. After cell recovery of 24 h, TCRγδ expression was restored and CD107a assays, in order to determine AIC and ADCC, were performed. When analyzing expanded γδ T cells in CD107a-degranulation assays a high baseline-expression of CD107a of 30–50% was observed (Figure 1D). Addition of CD19-specific constructs did not enhance expression of CD107a. TNFα expression of γδ T cells was marginally enhanced by N19-C16 and 4G7SDIE when incubated with target cell line NALM-6. IFNγ expression was slightly enhanced by N19-C16 when incubated with target cell line NALM-6.

### Correlation of CD16-expression by primary γδ T cells with ADCC by CD19-specific antibody constructs

**Figure 2:**
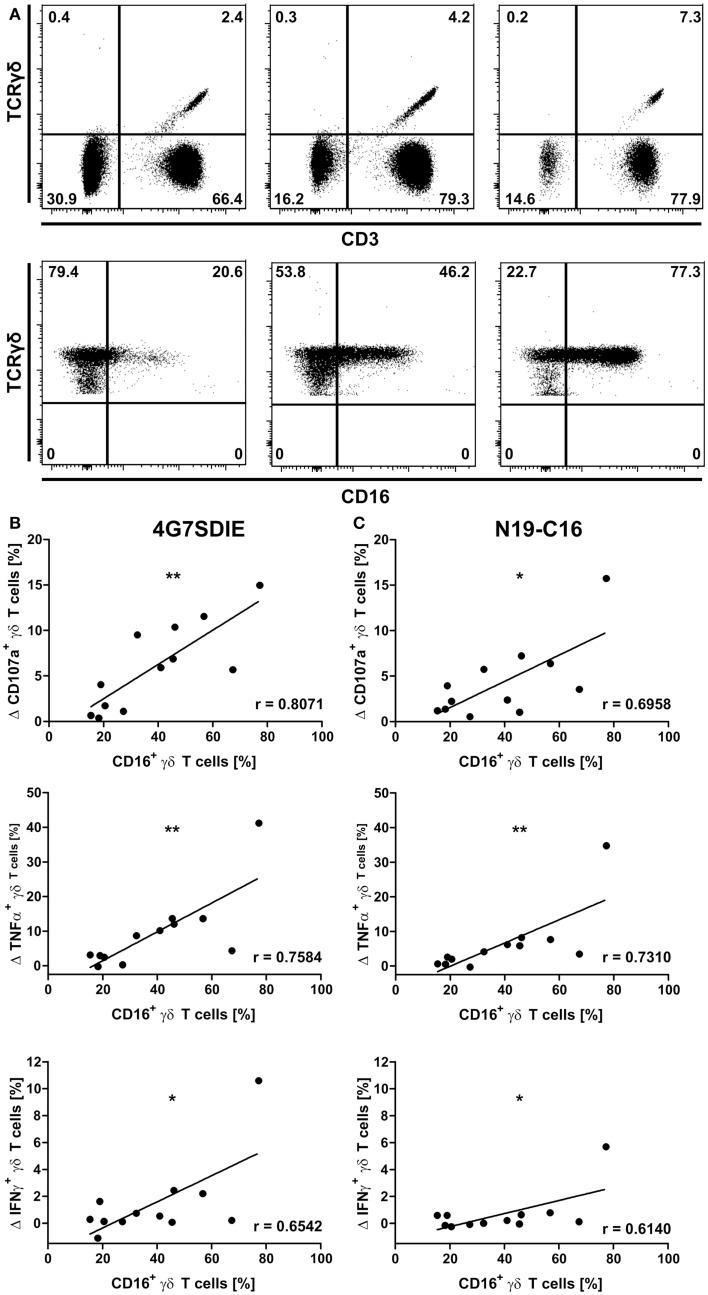
**Correlation of CD16-expression by γδ T cells and degranulation and cytokine production of antibody-stimulated primary γδ T cells**. CD16-expression of γδ T cells was assessed by flow cytometric analyses prior to functional assays. Gating hierarchy was: lymphocytes, single cells, viable cells, CD3^+^TCRγδ^+^, TCRγδ^+^CD16^+^. Gates were defined based on marker-negative populations within the viable cells. Three representative donors are shown. Same gating strategy was used for all samples **(A)**. PBMC were incubated with equal cell numbers of target cell lines NALM-6 and antibodies for 16 h. γδ T cell degranulation (CD107a) and cytokine production (TNFα and IFNγ) were detected on viable, single CD3^+^TCRγδ^+^ lymphocytes by extracellular and intracellular staining and flow cytometric analysis. Expression of CD107a, TNFα, and IFNγ induced by NALM-6 only was deducted from expression levels reached by incubation with NALM-6 and 4G7SDIE or N19-C16. PBMC were incubated with target cell line NALM-6 and medium, 1 μg/ml 4G7SDIE **(B)** and 1 μg/ml N19-C16 **(C)**, respectively. Statistical significance was assessed using Pearson’s correlation. Experiments were performed in triplicates.

CD16-expression of γδ T cells was not detected simultaneously in CD107a-degranulation assays due to technical limitations, which prohibited staining of CD16 when analyzing the CD19-specific antibody constructs. Hence, PBMC samples were stained for γδ T cell markers CD3 and TCRγδ as well as CD16 prior to CD107a-degranulation assays with 4G7SDIE and N19-C16 and cell line NALM-6 as described above (Figure 2A). Mean percentage of CD16-positive γδ T cells was 38.93% ± 20.43. Expression of CD107a, TNFα, and IFNγ induced by target cell line only was deducted from expression levels reached by incubation with NALM-6 and CD19-specific antibody constructs. A positive correlation between CD107a-, TNFα-, and IFNγ-expression by 4G7SDIE (Figure 2B) or N19-C16 (Figure 2C) stimulated γδ T cells and CD16-positive γδ T cells was observed.

### Assessment of cytotoxicity of expanded γδ T cells in 2 h-europium-TDA release assays

**Figure 3:**
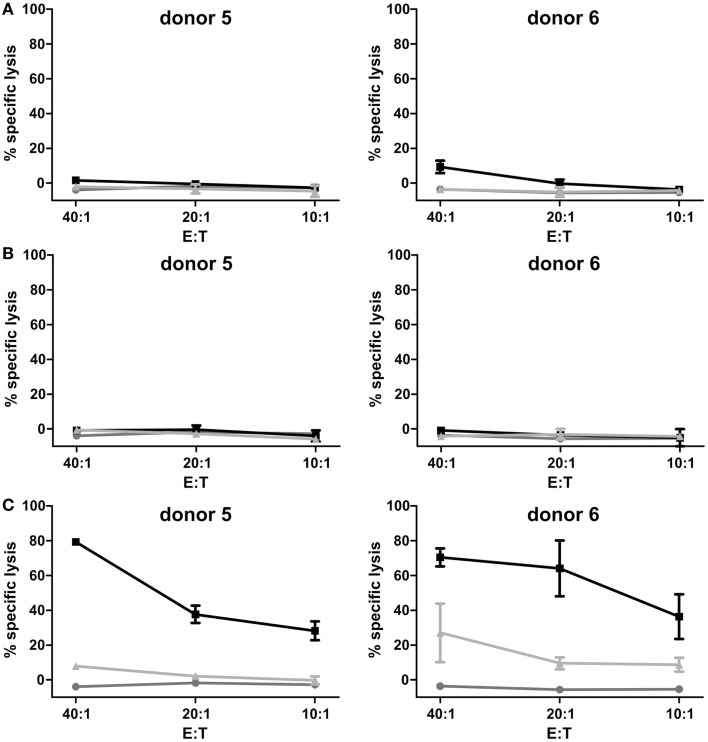
**Assessment of γδ T cell-mediated AIC and ADCC in 2 h-europium-TDA release assays**. Percentage of specific lysis was detected in europium-TDA release assays after co-incubation of 5000 labeled pediatric B-lineage ALL blasts per well with expanded, isolated and recovered γδ T cells at different E:T ratios (20:1–5:1) and antibodies for 2 h. The cytolytic activity after 2 h was calculated as the percentage of specific lysis [ = (experimental TDA release − spontaneous TDA release)/(maximum TDA release − spontaneous TDA release) × 100]. Two representative donors of four are shown. **(A)** γδ T cells (gray line), γδ T cells with 1 μg/ml control antibody (light gray line), and γδ T cells with 1 μg/ml CD19-specific Fc-optimized mAb 4G7SDIE (black line) were added, respectively. **(B)** γδ T cells (gray line), γδ T cells with 1 μg/ml control antibody (light gray line) and γδ T cells with 1 μg/ml CD19–CD16-bi-specific antibody construct N19-C16 (black line) were added, respectively. **(C)** γδ T cells (gray line), γδ T cells with 100 ng/ml control antibody (light gray line) and γδ T cells with 100 ng/ml CD19–CD3-bi-specific antibody construct N19-CU (black line) were added, respectively. Experiments were performed in triplicates. Error bars represent the standard deviation (SD).

After expansion of γδ T cells the TCRγδ^+^-cell population was isolated (purity 99.5% ± 0.62) and after cell recovery of 24 h cytotoxicity assays in order to determine AIC and ADCC were performed. First, cytotoxicity of isolated, expanded, and recovered γδ T cells was assessed in 2 h-europium-TDA release assays with pediatric B-lineage ALL blasts. No significant lysis of leukemic blasts by expanded γδ T cells and γδ T cells with CD19-specific antibody constructs 4G7SDIE and N19-C16 was observed in this end-point assay, respectively (Figures 3A,B). As positive control, 2 h-europium-TDA release assays with pediatric B-lineage ALL blasts, γδ T cells and a CD19–CD3 bi-specific antibody construct (N19-CU) were performed (Figure 3C). Albeit γδ T cells alone did not lyse pediatric B-lineage ALL blasts, combination of γδ T cells with N19-CU did greatly enhance lysis.

### Assessment of cytotoxicity of expanded γδ T cells in a label-free real-time assay (xCELLigence)

In order to test the suitability of impedance-based measurement of cell viability for monitoring γδ T cell-mediated AIC and ADCC by CD19-specific antibody constructs, CD19-expressing, adherent cells were required. Adherent breast adenocarcinoma cell line MCF-7 was stably transfected with transmembrane CD19. A CD19^+^ clone, expressing CD19 comparable to CD19 surface expression levels displayed by pediatric B-lineage ALL blasts, was selected by cell sorting (Figure S1 in Supplementary Material).

**Figure 4:**
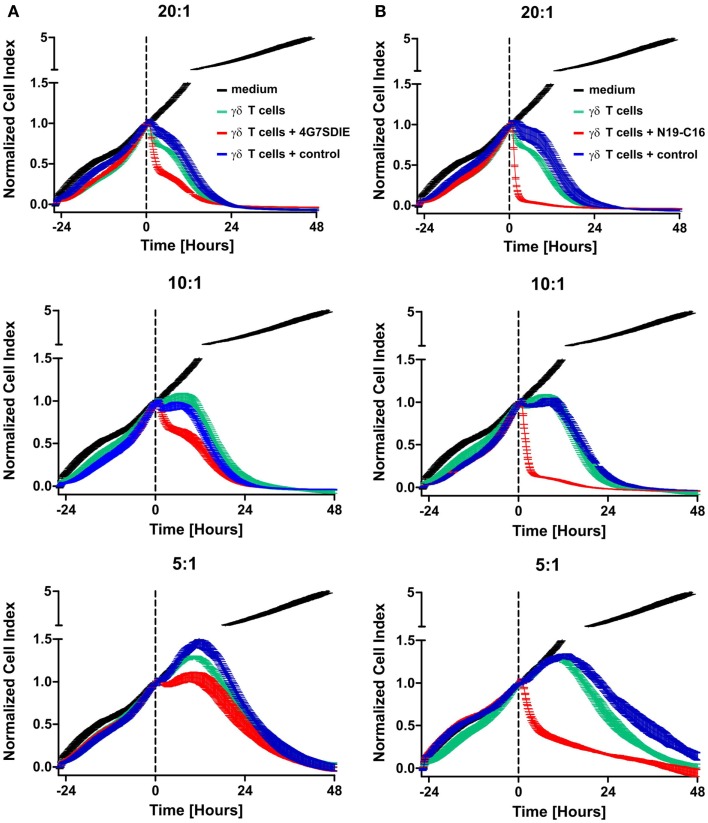
**Dynamic monitoring of expanded γδ T cell-mediated AIC and ADCC (xCELLigence assay)**. **(A,B)** MCF-7-CD19tm were seeded into 96-well E-plates at equal densities of 5000 cells per well. After cell attachment and expansion, expanded, isolated, and recovered γδ T cells at different E:T ratios (20:1–5:1) and antibodies were added at time-point *t*_0_ (indicated by the dashed line) Impedance at well bottoms was measured every 15 min for >48 h and normalized to baseline impedance values with medium only. Changes in impedance normalized to *t*_0_ are given as dimensionless normalized cell index (CI). One representative experiment, of four independent experiments performed, is shown. **(A)** Medium without effector cells (black line), γδ T cells (green line), γδ T cells with 1 μg/ml control antibody (blue line), and γδ T cells with 1 μg/ml CD19-specific Fc-optimized mAb 4G7SDIE (red line) were added, respectively. **(B)** Medium without effector cells (black line), γδ T cells (green line), γδ T cells with 1 μg/ml control antibody (blue line), and γδ T cells with 1 μg/ml CD19–CD16-bi-specific antibody construct N19-C16 (red line) were added, respectively.

**Figure 5:**
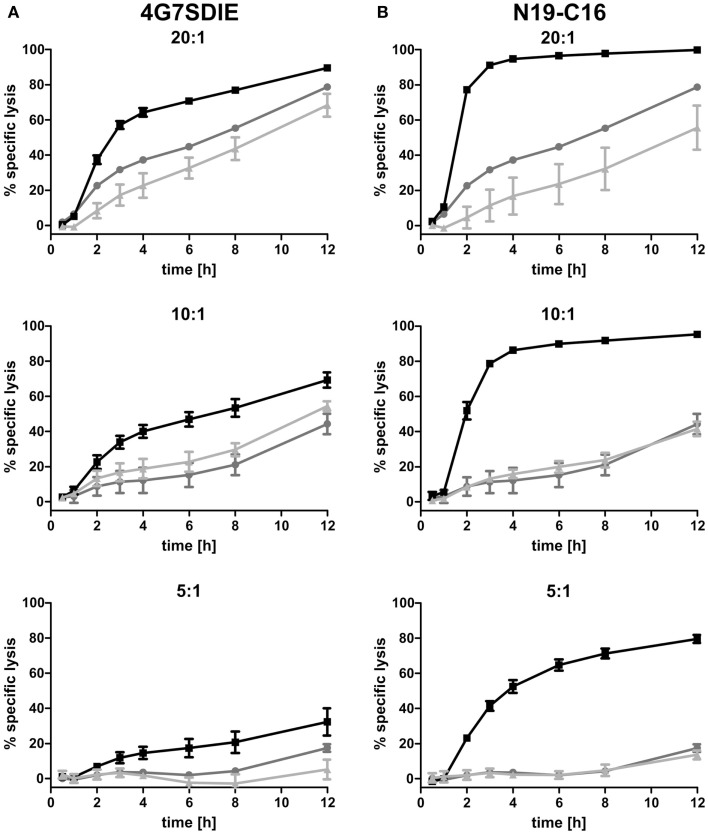
**Time-dependent cytolytic activity of expanded γδ T cells in xCELLigence assays**. **(A,B)** MCF-7-CD19tm were seeded into 96-well E-plates at equal densities of 5000 cells per well. After cell attachment and expansion, expanded, isolated, and recovered γδ T cells at different E:T ratios (20:1–5:1) and antibodies were added at time-point t_0_. Impedance at well bottoms was measured every 15 min for >12 h and normalized to baseline impedance values with medium only. The cytolytic activity at different time-points was calculated as the percentage of specific lysis (= [normalized CI_no effector cells_ − normalized CI_γδ T cells (with_ _antibody)_]/normalized CI_no effector cells_ × 100). One representative experiment, of four independent experiments performed, is shown. **(A)** γδ T cells (gray line), γδ T cells with 1 μg/ml control antibody (light gray line) and γδ T cells with 1 μg/ml CD19-specific Fc-optimized mAb 4G7SDIE (black line) were added, respectively. **(B)** γδ T cells (gray line), γδ T cells with 1 μg/ml control antibody (light gray line), and γδ T cells with 1 μg/ml CD19–CD16-bi-specific antibody construct N19-C16 (black line) were added, respectively.

MCF-7-CD19 transfected cells of the selected clone (MCF-7-CD19tm) were seeded on 96-well E-plates and after growing overnight expanded isolated and recovered γδ T cells were added in various effector to target ratios (E:T). Medium, control antibody, CD19-specific antibody constructs 4G7SDIE and N19-C16 were added at the same time-point, respectively. An E:T ratio-dependent cytolysis of target cells could be reproducibly monitored as decreasing impedance values in real-time, whereas medium controls were not affected in their growing displayed by continuously increasing normalized CI values (Figures 4A,B). Depending on E:T ratios, normalized CI values of MCF-7-CD19tm incubated with expanded γδ T cells reached baseline, displaying a target cell lysis of 100%, after 24–48 h (Figures 4A,B). Addition of CD19-specific antibody constructs 4G7SDIE and N19-C16 greatly enhanced the reduction of normalized CIs of MCF-7-CD19tm cells. Specific lysis of target cells was calculated for different time-points (Figures 5A,B). At E:T 20:1 specific lysis was increasing rapidly over time and reached 79% after 12 h whereas lysis with E:T 10:1 and E:T 5:1 was increasing less rapidly over time, reaching 44 and 17% after 12 h, respectively. Addition of CD19-specific antibody constructs 4G7SDIE and N19-C16 greatly enhanced the specific lysis of MCF-7-CD19tm cells. Depending on the time-point to observe AIC and ADCC, differences between γδ T cells alone (AIC) and γδ T cells with CD19-specific antibody constructs (ADCC), were more or less pronounced (Figures 5A,B). Greatest differences of AIC and ADCC were at 4 h (20:1) and 8 h (10:1 and 5:1) for 4G7SDIE and 4 h (20:1), 6 h (10:1) and 8 h (5:1) for N19-C16, respectively.

## Discussion

Albeit tremendous improve in outcome of pediatric patients with B-lineage ALL over the last decades, prognosis for primary refractory or relapsed patients remains poor. Since immunotherapeutic therapy with chimeric antigen receptor-modified T cells with specificity for CD19 have been successful in pediatric patients with B-lineage ALL, further immunotherapeutic strategies are emerging (40, 41).

Due to their features including non-MHC restriction, cytotoxicity against hematological malignancies and capacity for ADCC, γδ T cells are promising effector cells for immunotherapy of pediatric B-lineage ALL during early post-transplant phase and phase and cell-based immunotherapy after SCT.

In this study, we evaluated the combination of primary and expanded human γδ T cells with CD19 antibodies for immunotherapy of pediatric B-lineage ALL and established an label-free method, facilitating the long-term and real-time monitoring of γδ T cell-mediated antibody-independent (AIC) and ADCC.

Indirect cytotoxicity and cytokine production of primary γδ T cells was analyzed in CD107a-degranulation assays and ICS. A significantly increased expression of degranulation marker CD107a and cytokine production by γδ T cells was observed when target cells were incubated with PBMC and CD19 antibody constructs. However, expanded, isolated, and recovered γδ T cells were highly CD107a-positive and CD107a expression was not further enhanced by CD19 antibody constructs. In contrast to Fc-optimized mAb 4G7SDIE, no activation of γδ T cells was induced by parental non-optimized antibody χ4G7. This indicates that a strong activation may be needed for γδ T cell-mediated ADCC. Measurement of CD107a-degranulation combined with ICS is a suitable method for analyzing ADCC capacity of untouched, primary γδ T cells without the need to isolate γδ T cells. For expanded γδ T cells direct cytotoxicity assays seem favorable. For Rituximab it has been shown that γδ T cell ADCC is driven by CD16 (42). We observed a positive correlation between percentages of CD16-positive γδ T cells and degranulation and cytokine production upon CD19-specific antibody stimulation. This finding indicates that CD107a-, TNFα-, and IFNγ-positive γδ T cells belong to the CD16^+^ subset of the γδ T cell pool. Inhibiting CD16-mediated signaling by using a blocking antibody or removing CD16^+^ γδ T cells from the γδ T cell population may be possible approaches to further underline this hypothesis. It has been shown, that the percentage of CD16^+^ cells in the γδ T cell pool can be elevated in response to pro-inflammatory cytokines IL-2, IL-15, and IL-21 (43, 44). Enhancing CD16-expression of γδ T cells *in vivo* and in clinical large-scale expansion protocols, respectively, may be approaches to advance combined immunotherapy with therapeutic antibodies. Examining the effect of these cytokines on γδ T cell-mediated ADCC would be particularly interesting as it has been shown that NK cell-mediated ADCC is enhanced by IL-2, IL-15, and IL-21 as well (7, 45, 46).

CD107a-degranulation assays measure the cytolytic capacity of effector cells but lack detection of direct cytolysis of target cells. However, considering the low frequencies of primary γδ T cells, isolation of sufficient numbers of γδ T cells for direct cytotoxicity assays may be impracticable. Furthermore, analysis of ADCC of untouched primary γδ T cells in direct cytotoxicity assays, requiring isolation of cells, is currently challenging as, to our knowledge, no cell isolation kits are commercially available, which allow isolation of γδ T cells without depleting CD16^+^ γδ T cells. γδ T cells were expanded after a protocol, using reagents in pharmaceutical quality, making a possible clinical translation achievable. AIC and ADCC of expanded, isolated and recovered γδ T cells was analyzed in 2 h-europium-TDA assays. No lysis of leukemic blasts by expanded γδ T cells alone and with 4G7SDIE and N19-C16 was measured, respectively. When expanded γδ T cells were strongly activated by a CD19–CD3-recruiting bi-specific antibody construct (N19-CU), strongly enhanced lysis was observed. Unlike CD16, which was expressed by 26.55% ± 6.02 of the expanded γδ T cells, CD3 is expressed by every γδ T cell. Hence, activation of a significantly greater number of γδ T cell can be achieved with antibodies recruiting CD3-positive cells rather than CD16-positive cells. This end-point assay is feasible for detecting cytotoxicity of expanded γδ T cells against leukemic blasts. However, potent and rapid activation of expanded γδ T cells seems to be required. AIC and ADCC of expanded γδ T cells may be obliterated due to the short end-point of the assay, which is limited by time-dependent increase of spontaneous release of TDA by labeled target cells. Furthermore, no conclusions to lysis kinetics can be drawn.

Due to the mentioned limitations of the end-point assay, AIC and ADCC of γδ T cells were detected in real-time cytotoxicity assays (xCELLigence). One drawback of this method is the requirement of adherent target cells. Since primary leukemic blasts as well as common leukemic cell lines are suspension cells, a CD19-expressing target cell line was generated. In consideration of that the generated cell line MCF-7-CD19tm expressed comparable CD19 surface levels to leukemic blasts and commonly used cell lines, we hypothesize that results obtained in this assay allow conclusions regarding ADCC by CD19 antibody constructs and γδ T cells against CD19-expressing leukemic blasts. Significant lysis of target cell line MCF-7-CD19tm by expanded γδ T cells was observed and increased over time. CD19 antibody constructs 4G7SDIE and N19-C16 greatly enhanced the specific lysis of MCF-7-CD19tm cells by expanded γδ T cells. Notably, maximal cytotoxicity of expanded γδ T cells was delayed beyond 24 h and time-points of reaching maximal cytolysis varied for expanded γδ T cells alone (AIC) and expanded γδ T cells with CD19 antibody constructs (ADCC). Depending on the time-point considering AIC and ADCC, differences between γδ T cells alone (AIC) and γδ T cells with CD19 antibody constructs (ADCC), were more or less pronounced. These observations underline the importance of prolonged incubation times in cytotoxicity assays with expanded γδ T cells and monitoring lysis kinetics by real-time measurement of AIC and ADCC.

In summary, we show that untouched primary as well as expanded γδ T cells mediate ADCC with CD19 antibody constructs against various targets. Thus, combination of the presented antibody constructs with primary as well as expanded γδ T cells exhibit promising immunotherapeutic approaches that require clinical evaluation. Notably, assays assessing AIC and ADCC of γδ T cells have some limitations and should be choosen deliberately. The assessment of CD107a and cytokine expression is a feasible method for assessing cytotoxicity of untouched primary γδ T cells rather than expanded γδ T cells. Europium-TDA release assays are feasible for expanded γδ T cells but AIC and ADCC of γδ T cells can be obliterated due to the limited end-point of the assay and no firm conclusions to lysis kinetics may be drawn. The xCELLigence system allows to measure lysis kinetics of γδ T cells over prolonged periods of time and enables the detection of both AIC and ADCC of expanded γδ T cells against adherent target cells.

## Author Contributions

Ursula Jördis Eva Seidel contributed to the conception and design of the work, development of methodology, acquisition, analysis and interpretation of data, and to the drafting of this article. Fabian Vogt contributed to the development of methodology and interpretation of data and critical revision of this article. Ludger Grosse-Hovest contributed to the development of methodology, acquisition, analysis and interpretation of data, and critical revision of this article. Gundram Jung contributed to the interpretation of data and critical revision of this article. Rupert Handgretinger contributed to the conception and design of the work, interpretation of data, and critically revised this article for the final approval of the submitted version. Peter Lang contributed to the conception and design of the work, interpretation of data, and critical revision of this article.

## Conflict of Interest Statement

The authors declare that the research was conducted in the absence of any commercial or financial relationships that could be construed as a potential conflict of interest.

## Supplementary Material

The Supplementary Material for this article can be found online at http://www.frontiersin.org/Journal/10.3389/fimmu.2014.00618/abstract
