# Supplementary material for: γδ T Cell-Mediated Antibody-Dependent Cellular Cytotoxicity with CD19 Antibodies Assessed by an Impedance-Based Label-Free Real-Time Cytotoxicity Assay
Source: Front Immunol. 2014 Dec 2;5:618. doi: 10.3389/fimmu.2014.00618 (PMC4251440; doi:10.3389/fimmu.2014.00618)
Supplement: Supplementary file 1 [file Image_1.PDF]

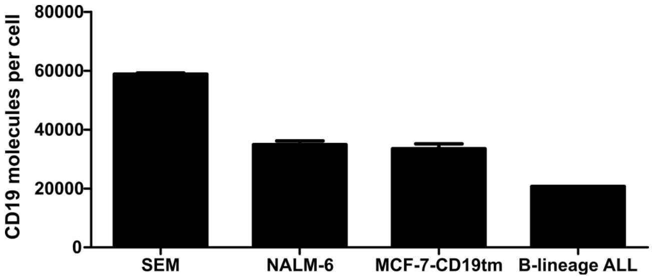

Supplementary figure 1. CD19 expression on cell lines and pediatric B-lineage ALL blasts. Cell lines and blasts from a pediatric common-ALL patients were incubated with murine 4G7 (5 µg/ml), washed and bound antibody was detected by indirect immunofluorescence staining and flow cytometry. Leukemic cells were gated on $CD10^{+}CD34^{+}$ cells. Molecules per cell were calculated by comparison with calibrated beads (QIFIKIT).
